# Tyrosine and Tryptophan vibrational bands as markers of kidney injury: a renocardiac syndrome induced by renal ischemia and reperfusion study

**DOI:** 10.1038/s41598-021-93762-z

**Published:** 2021-07-22

**Authors:** Gabrielle Nepomuceno, Carolina Victoria Cruz Junho, Marcela Sorelli Carneiro-Ramos, Herculano da Silva Martinho

**Affiliations:** Universidade Federal do ABC, Centro de Ciências Naturais e Humanas, Av. dos Estados, 5001, Santo André, SP 09210-580 Brazil

**Keywords:** Cardiology, Cardiovascular biology, Interventional cardiology, Cardiovascular biology, Kidney

## Abstract

Renal injury caused by renal ischemia and reperfusion strongly influences heart morphology, electrophysiology, and redox unbalance. The so-called cardiorenal syndrome is an important class of dysfunction since heart and kidneys are responsible for hemodynamic stability and organ perfusion through a complex network. In the present work we investigate the vibrational spectral features probed by Fourier-Transform Raman (FT-Raman) spectroscopy due to physiological alterations induced by renal ischemic reperfusion aiming to detect molecular markers related to progression of acute to chronic kidney injury and mortality predictors as well. C57BL/6J mice were subjected to unilateral occlusion of the renal pedicle for 60 min and reperfusion for 5, 8, and 15 days. Biopsies of heart and kidney tissues were analyzed. Our findings indicated that cysteine/cystine, fatty acids, methyl groups of Collagen, α-form of proteins, Tyrosine, and Tryptophan were modulated during renal ischemia and reperfusion process. These changes are consistent with fibroblast growth factors and Collagen III contents changes. Interestingly, Tyrosine and Tryptophan, precursor molecules for the formation of uremic toxins such as indoxyl sulfate and p-cresyl sulfate were also modulated. They are markers of kidney injury and their increase is strongly correlated to cardiovascular mortality. Regarding this aspect, we notice that monitoring the Tyrosine and Tryptophan bands at 1558, 1616, and 1625 cm^−1^ is a viable and and advantageous way to predict fatality in cardiovascular diseases both “in vivo” or “in vitro”, using the real-time, multiplexing, and minimally invasive advantages of FT-Raman spectroscopy.

## Introduction

The cardiorenal syndrome (CRS) is a systemic condition which heart and kidneys interact in a way that an acute or chronic injury caused in one generates a pathology in the other organ^1^. It could be classified into five main types. Types 1 and 2 correspond to the cardiorenal syndrome itself, when some injury in heart leads to a kidney injury as consequence; types 3 and 4 (also called renocardiac syndrome) an injury in kidney that leads to a heart injury; and type 5 when a systemic condition leads to a simultaneous heart and kidney injury (e.g., diabetes and sepsis)^1^. All types share the inflammation as key cause^2^.

It is already known that CRS type 3 can induce cardiac injuries, being the most studied by our group, the cardiac hypertrophy. It has already been observed that the renal injury caused by renal ischemia and reperfusion (IR) is capable to change heart morphology^3^, electrophysiology^4^ and redox unbalance with participation of immune system^5^. Another remarkable aspect refers to the increasing pieces of evidence about the relationship between uremic toxins and cardiovascular diseases^6^. Besides, the main role of kidney is blood filtration eliminating waste products resulting from the metabolism (e.g. urea, creatinine, uric acid, indoxyl sulfate). Kidney failure can be characterized by an accumulation of toxic compounds, including some metabolites which have a well-documented toxicity referred as uremic toxins (UT). Despite limited available knowledge concerning the molecular mechanisms which lead to cardiovascular diseases, many reported works have already indicated that cardiovascular mortality is correlated with the increase of uremia and its toxic effects^6^.

Biophotonics techniques as Raman spectroscopy have been successfully employed to probe biological samples since they provide multiplexing quantitative and qualitative pieces of information at the molecular level even when gated by subtle changes in a sample^7^. This technique has been used in cells, tissues and biofluids studies presenting valuable findings related to pathologies^8–10^. Outstanding results concerning cardiac tissues were also reported on literature (see, e.g. ^11^.). For example, Nishiki-Muranish et al.^12^ analyzed Raman spectra of myocardial infarction and its repair in rats. They found that the course of myocardial infarction and its repair could be recognized by spontaneous Raman spectroscopy based on chemical changes in myocardial tissues. Normal, necrotic, granulation, and fibrotic tissues were discriminated with cross-validated sensitivities of 99.3, 95.3, 96.4, and 91.3% and specificities of 99.4, 99.5, 96.5, and 98.3% respectively. The authors shown that spontaneous Raman spectroscopy combined with p*artial least squares-discriminant analysis* (PLS-DA) could be used as a novel label-free method of evaluating myocardial infarction and its repair.

Yamamoto et al.^13^ identified key signatures of Raman spectra for the evaluation of myocardial viability by evaluating the infarct border zone myocardium. Tissues were excised from five patients under surgical ventricular restoration. They were able to obtain a prediction model to differentiate the infarcted myocardium from the non-infarcted myocardium by applying PLS-DA to the Raman spectra. Ohira et al.^14^ used Raman spectroscopy for evaluate myocardial *ischaemia* especially during early ischaemic phase. They obtained spontaneous Raman spectra of the sub-epicardial myocardium in the Langendorff-perfused rat heart upon 532-nm excitation before and during the “stopped-flow,” global ischaemia. They showed that sequential measurements of the band intensities at 750 and 1127 cm^−1^ enabled early detection of the myocardial *ischaemia* based on the mitochondrial functions.

Notwithstanding the relevance of the cited works, applications of Raman spectroscopy in the context of CRS are absent, to the best of our knowledge. The focus of this study was to characterize the vibrational pattern of molecules on cardiac and renal tissues aiming to find molecular markers relating progression of acute to chronic kidney injury related to CRS. Of special interest is probe mortality predictors as uremic toxins (UT). The spectral variations induced by reperfusion were investigated and their biochemical correlations presented and discussed.

## Materials and methods

### Animal model of renal ischemia and reperfusion (IR)

The experiments were carried out following the Brazilian federal law nº 11.794 of 2008 and approved by the licensing committee “Comissão de Ética em Uso de Animais da UFABC (CEUA)” of the Universidade Federal do ABC, Brazil (protocol number 2728130318). We also state that all ARRIVE (Animal Research: Reporting of In Vivo Experiments) recommendations were followed. Male C57BL/6 mice, aged between 6 and 8 weeks weighing between 20 and 25 g, were used. All animals were housed in collective cages, containing a maximum of five animals, with a 12-hr light/dark artificial cycle, at a constant ambient temperature of 25 $^\circ$C, with water and food supplement *ad libitum*.

The protocol of IR induction was described elsewhere^5^. The animals were submitted to anesthesia with xylazine and ketamine intraperitoneally at a single dose of 10 mg/kg and 100 mg/kg of weight, respectively diluted in 0.9% saline solution. Access to the renal pedicle was made through the opening of abdominal cavity. All internal organs were then bounced in hydrophilic gases. After locating the left renal pedicle of the animal, all adipose tissue around region was removed. Then the left pedicle was isolated using forceps and occluded using microvascular clips. After placing the clamp on the renal pedicle, an immediate change in kidney color could be observed due to occlusion of the local circulation, indicating the efficiency in inducing renal ischemia. Animals were clamped and kept in a thermal blanket for 60 min. Then the clamps were removed, and viscera were replaced in the abdominal cavity with the use of flexible nails. The peritoneum and skin sutured using a 4/0 silk thread. After the surgical process, the animals were kept under reperfusion for 8 and 15 days.

Mice were divided into three groups: Sham (those under surgical process, except the occlusion of the renal pedicle; control; N = 3 animals), 8D (those under surgical procedure of occlusion of the left renal pedicle for 60 min and reperfusion for 8 days; N = 3 animals), and 15D (those under surgical procedure of occlusion of the left renal pedicle for 60 min and reperfusion for 15 days, N = 3 animals). After reperfusion time, the animals were euthanized by blood extraction through the inferior *vena cava*. The heart and left kidneys were collected for biopsies. The heart myocardium was collected removing the left ventricle wall while the kidney samples were collected removing the renal cortex. Biopsies of heart myocardium andrenal cortex from kidney were used for spectroscopy analysis. They were snap frozen and stored in −80° C ultrafreezer after surgical procedure.

### Fourier-Transform Raman (FT-Raman) spectroscopy

Left kidney and heart biopsies were kept under physiological serum prior vibrational spectroscopy measurements. The FT-Raman Multiram spectrometer (Bruker Optics, Germany) operating at 1064 nm (laser  Cobolt Rumba series, from Cobolt AB, Sweden) was used for acquisition of spectra. Each biopsied tissue was previously thawed at room temperature in saline solution (0.9% NaCl) at the time of use in the experiment and placed in an aluminum sample holder. Measurements were done at three different points (27 spectra per group) using 32 cycles of scans and 200 mW of laser power (laser spot size of diameter of 1 mm). Signs of sample degradation were observed for longer acquisition times and greater laser powers.

### Statistical analysis

#### Principal components analysis (PCA)

The classical Principal Components Analysis (PCA)^15^ was performed on mean centered raw data to extract outliers and identify possible experimental bias. All spectral analysis steps were implemented in the ChemSpec vignette available in the software R^16^. Outliers where identied using the *Q* and *T*^*2*^ Hotelling’s statistics. The *Q* statistics indicates how well each observation matches the PCA model and *Q-residuals* measures the residual between a sample and its projection into the factors retained in the model. Large residual outliers can be detected by inspection of *Q* residuals. On the other hand, Hotelling's *T*^*2*^ value represents a measure of the variation in each sample within the model indicating how far each sample is from the center (scores = 0) of the model. It is a quantifier for scores outliers. The *T*^*2*^ Hotelling’s versus *Q-*residuals (reduced) plot were inspected for heart and kidney groups.

#### Partial least squares—discriminant analysis (PLS-DA)

All spectra were pre-processed to become comparable for the statistical analysis. The baseline was corrected using the least-squares polynomial curve fitting method as described by Lieber and Mahadevan-Jansen^17^. All spectra were normalized to mean and scaled using Pareto’s scaling^18^.

Then PLS-DA analysis was performed. PLS is a multivariate supervised method that uses linear regression of original variables to predict the class membership (Sham, 8D, 15D for heart and kidney groups). In our case the PLS regression was performed using the *plsr* function provided by R pls package^16,19^. The classification and cross-validation were performed using the corresponding wrapper function using the caret package^19^. A permutation test was performed to assess the performance of class discrimination. In each permutation, a PLS-DA model was built between the data and the permuted class labels using the optimal number of components determined by leave-one-out cross validation for the model based on the original class assignment. The class discrimination performance was measured using *classification accuracy*, *R*^2^, and Q^2^ parameters. The first one is based on prediction accuracy. The R^2^ parameter is the “goodness of fit” or explained variation which is based on the ratio of the between group sum of the squares and the within group sum of squares. On the other hand, *Q*^2^ is the “goodness of prediction”, or predicted variation, calculated from cross validation. In each round, the predicted data are compared with the original data, and the sum of squared errors is calculated being then summed over all samples (Predicted Residual Sum of Squares or PRESS). For convenience, the PRESS is divided by the initial sum of squares and subtracted from 1 to resemble the scale of the *R*^2^. Good predictions will have low PRESS or high *Q*^2^ while negative *Q*^2^ means that model is not at all predictive or is overfitted^20–22^.

Two quantifiers were used to measure the vibrational band frequency importance in PLS-DA model. The first, Variable Importance in Projection (VIP) is a weighted sum of squares of the PLS loadings taking into account the amount of explained spectral intensity-variation in each dimension. The other importance measure is based on the weighted sum of PLS-regression. The weights are a function of the reduction of the sums of squares across the number of PLS components. For multiple-group analysis, the same number of predictors will be built for each group and the average of the feature coefficients were used to indicate the overall coefficient-based importance.

## Results

**Figure 1:**
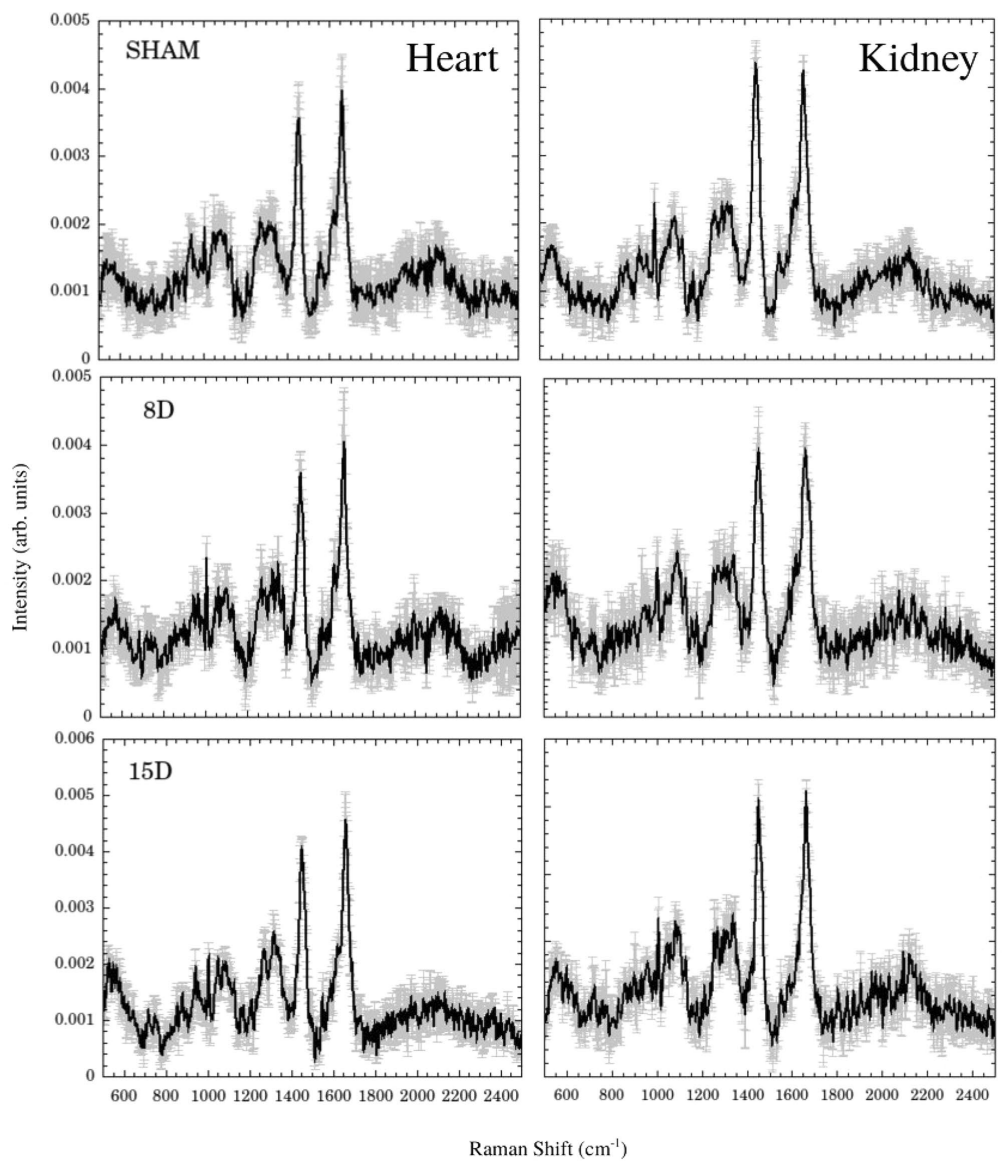
Average Raman spectra (black line) for heart (left) and kidney (right) tissues for SHAM, 8D, and 15D groups. The vertical gray lines are the standard deviation.

Figure 1 shows the Raman spectra for heart (left) and kidney (right) tissues groups (Sham, 8D, and 15D classes) in the 800–2500 cm^−1^ spectral window. Black lines are the average spectra while the gray vertical lines are the standard deviation of intensities. At first glance, bands around 540, 1100, 1300, 1450, 1650, and 2100 cm^−1^ dominates the spectra. They are associated to stretching of S–S in cysteine amino acid, stretching of C–C in lipids, twisting of CH_2_ in collagen and phospholipids, bending modes of CH_3_ in lipids and amino acids side chains, Amide I vibration of proteins, and oxidation of fatty acids, respectively^18–21^. Assignment for the relevant vibrational bands is presented on Table 1.

**Table 1:** Assignment of vibrational modes, based on refs.^18–21^.

Wavenumber (cm^−1^)	Assignment
520–530	S–S disulfide stretching in (cystine or cysteine amino acids
545	ν(S–S) trans-gauche-trans (aminoacid cysteine)
555	ν(S–S) trans-gauche-trans (aminoacid cysteine)
625	C–C twisting mode of phenylalanine
770	Ring breathing of tryptophan
1317	Guanine (B,Z-marker)
1336	Guanine
1346	Glucose
1432	Deoxyribose in B or Z DNA conformation
1442	CH_2_ bending mode (fatty acids)
1452	Bending modes of methyl groups (collagen)
1461	δCH_2_ , Disaccharides
1471	C=N stretching
1481	Amide II
1558	Tryptophan
1606	Cytosine (NH_2_ )
1616	C=C (Tyrosine or tryptophan)
1625	Tryptophan
1645	Amide I (α-helix)
1654	Amide I (α-helix of collagen)
1664	Amide I (of collagen)
1799	C=O lipids
2100	Oxidation of fatty acids

**Figure 2:**
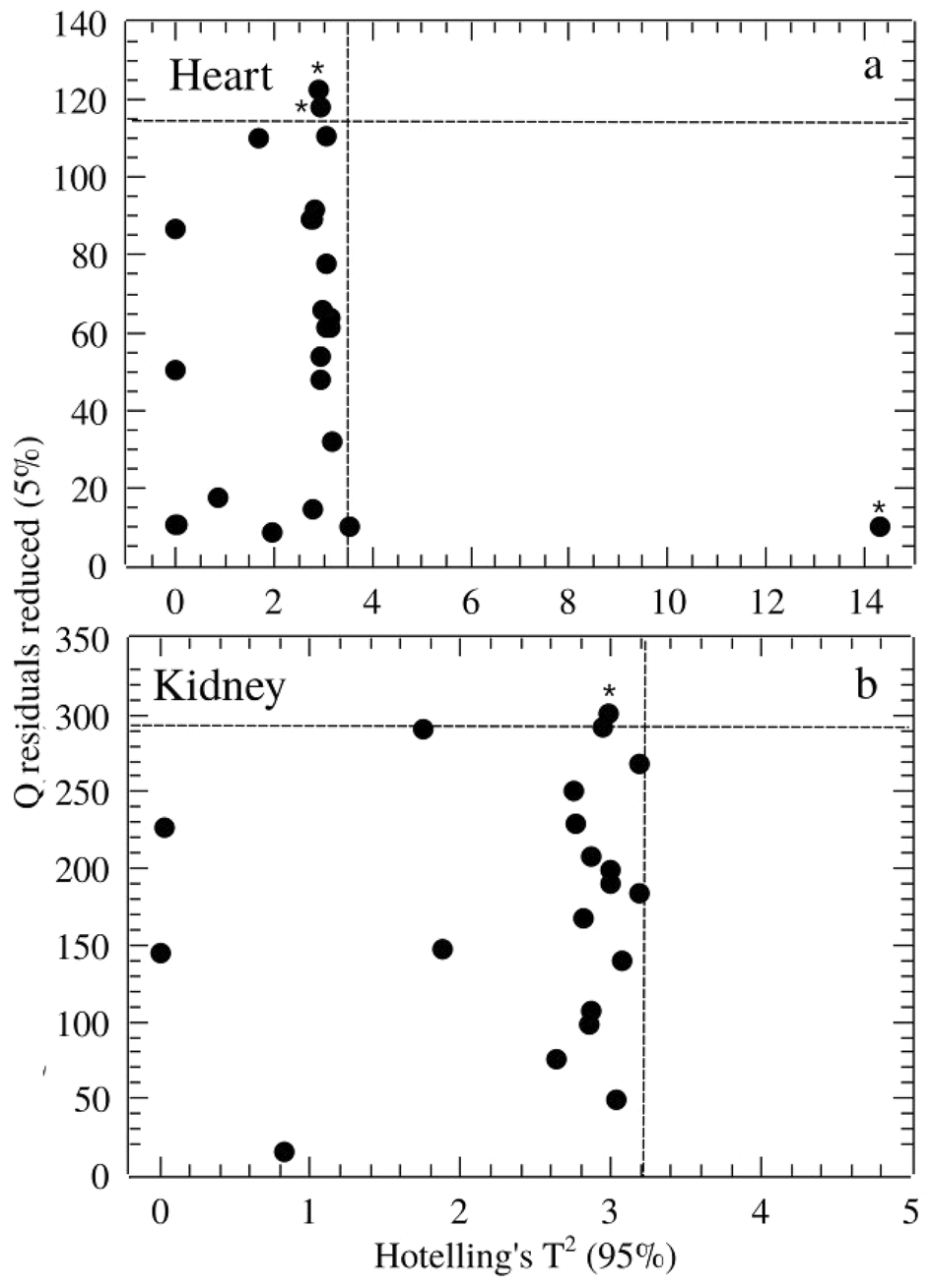
Outliers identification by inspection of Q residuals (reduced) versus Hotelling’s T^2^ plot for for heart (**a**) and kidney (**b**) groups. Outliers were indicated by “*”. Dashed lines horizontal and vertical lines represents confidence limit of 95% (Hotelling’s T^2^) and 5% (Q residuals), respectively.

Before processing and statistical analysis, a quality check evaluation was performed on raw spectral data to identify anomalous spectra, outliers and/or biased patterns. The PCA was computed on mean-centered raw data and the *Q* residuals (reduced) versus Hotelling’s *T*^2^ plot^21,22^ was checked (Fig. 2) in order to find residuals (*Q*) and scores (Hotelling’s *T*^2^) outliers. Data out of the confidence limits of 95% for scores and 5% for residuals were considered outliers (indicated by “^*^” in Fig. 2) and removed in the further analysis.

**Figure 3:**
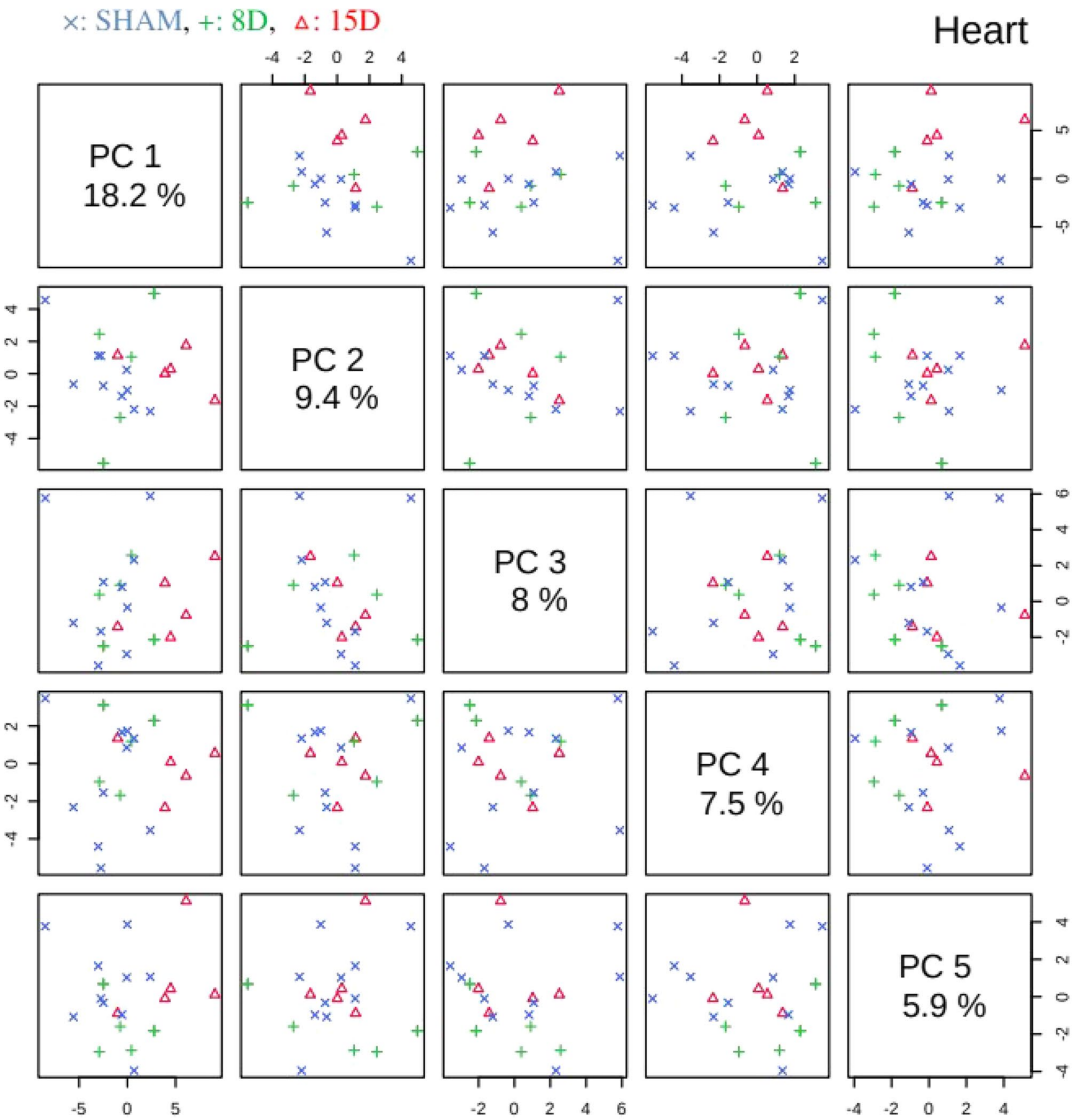
Pairwise score plots between the selected PLS-DA components for heart group. The explained variance of each component is shown in the corresponding diagonal cell. (x: SHAM, + : 8D, $\Delta$: 15D).

**Figure 4:**
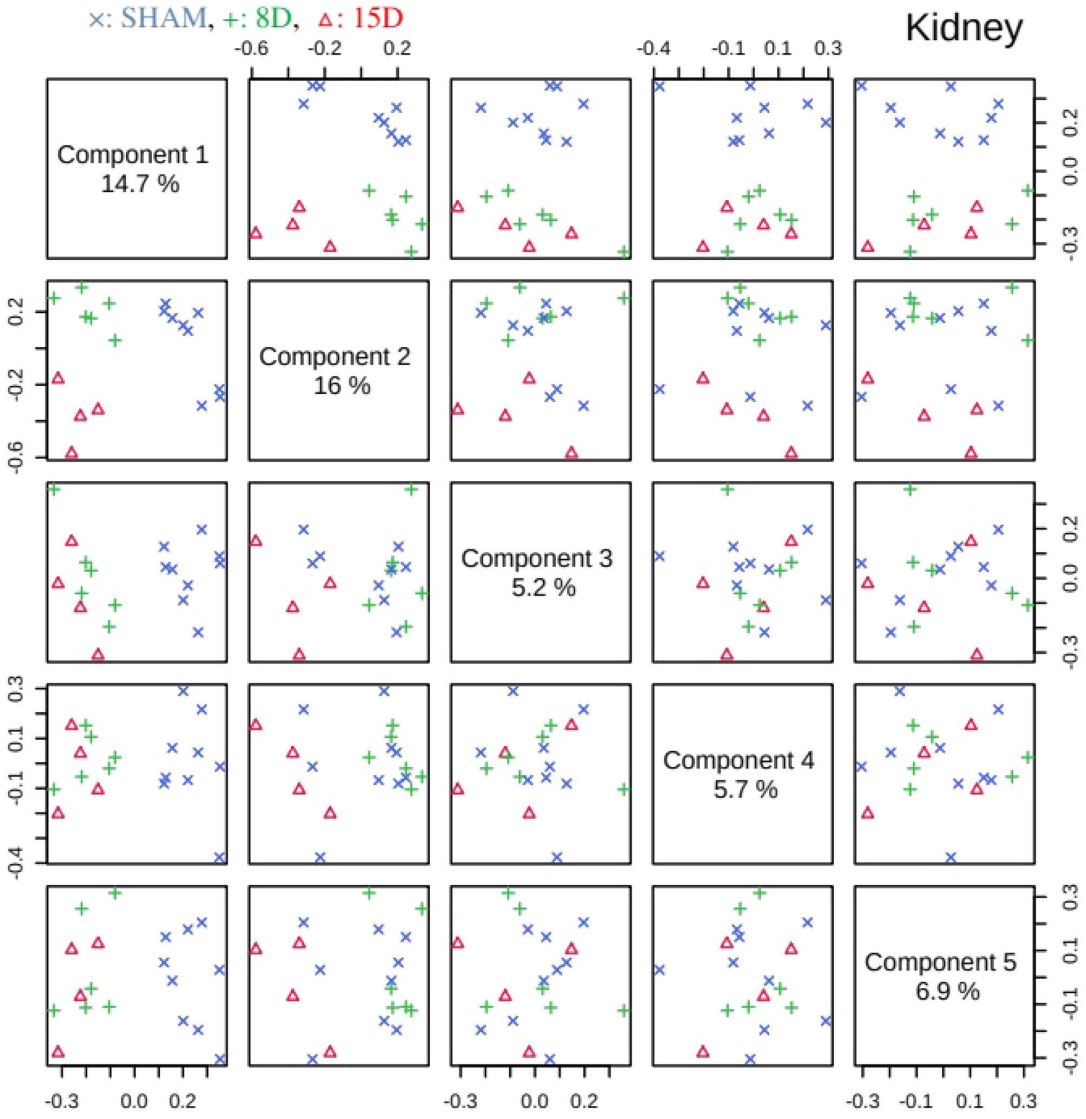
Pairwise score plots between the selected PLS-DA components for kidney group. The explained variance of each component is shown in the corresponding diagonal cell. (x: SHAM, + : 8D, $\Delta$: 15D).

**Figure 5:**
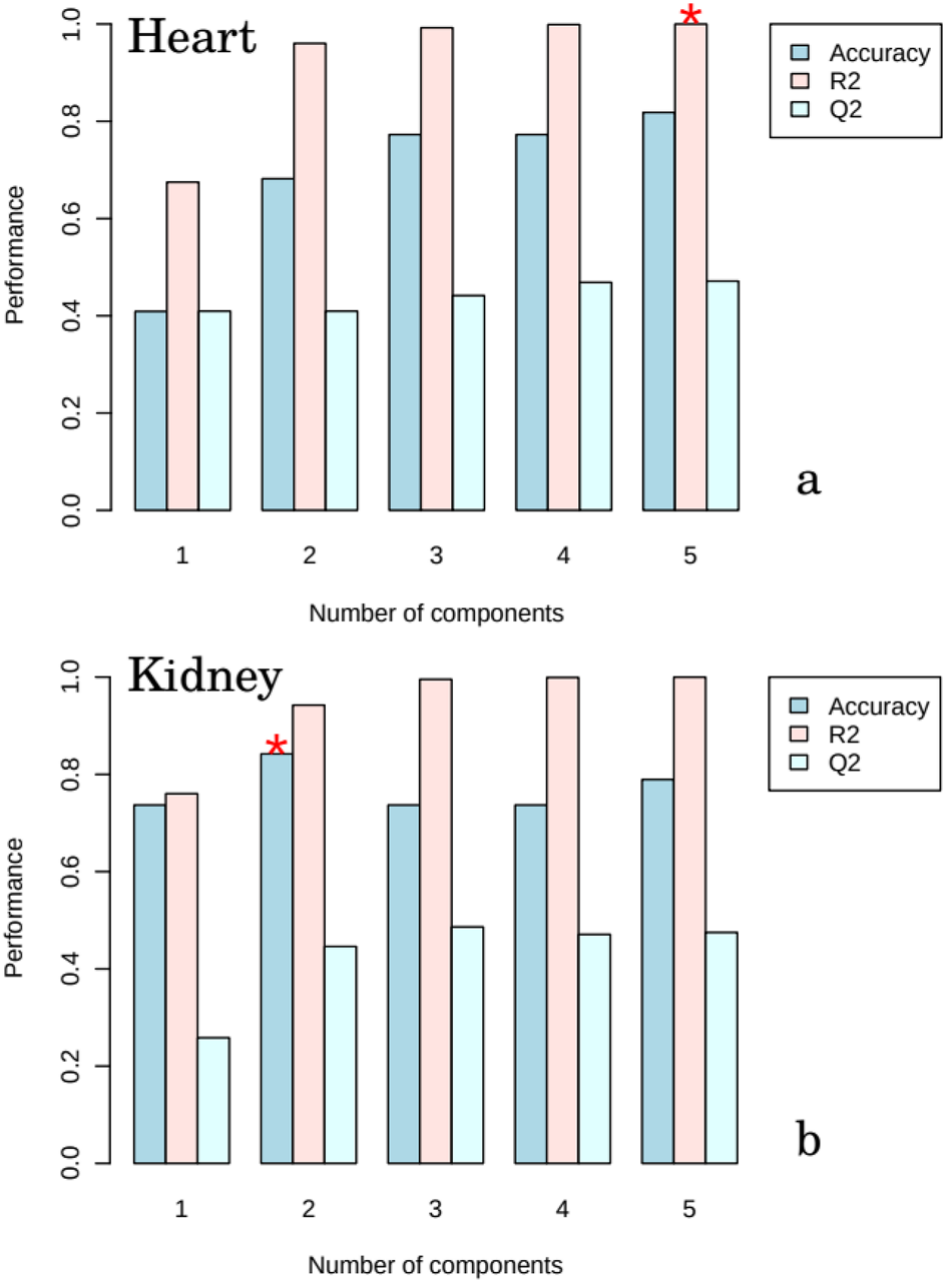
PLS-DA classification performance using different number of components for heart (**a**) and kidney (**b**) following Accuracy, R^2^ and Q^2^ criteria. The red star indicates the best classifier.

PLS-DA analysis was then performed on processed, normalized and scaled spectra. The optimal number of factors was determined by cross-validation after inspection of accuracy, R^2^, and Q^2^. Figures 3 and 4 present the pairwise scores up to 5th PC (5.9% of explained variance) for heart and kidney groups, respectively. For heart, at first glance combinations including PC1 are prone to discriminate 15D class of samples from the others. Those including PC5 tends to better classify 8D samples. In fact, the best PLS-DA classification performance for heart was observed considering 5 components (Fig. 5a). In this case the observed accuracy was 82% while *R*^2^ = *1.Q*^2^ appeared to be constant around 0.45 for all components. For kidney (Fig. 4) the combination PC1 and PC2 clearly discriminates the three experimental groups in accordance with the classification performance (Fig. 5b). The best accuracy was observed using 2 components (85%) while *R*^2^ = 0.98 and *Q*^2^ = 0.45. The high value of *R*^2^ indicates that models have a high predictive accuracy. In practice, it is difficult to give a general limit that corresponds to a good predictability since this strongly depends on the properties of the dataset. Since Q^2^ > 0.40 we can consider that models have a good predictive power as usually considered for biological samples^23^.

**Figure 6:**
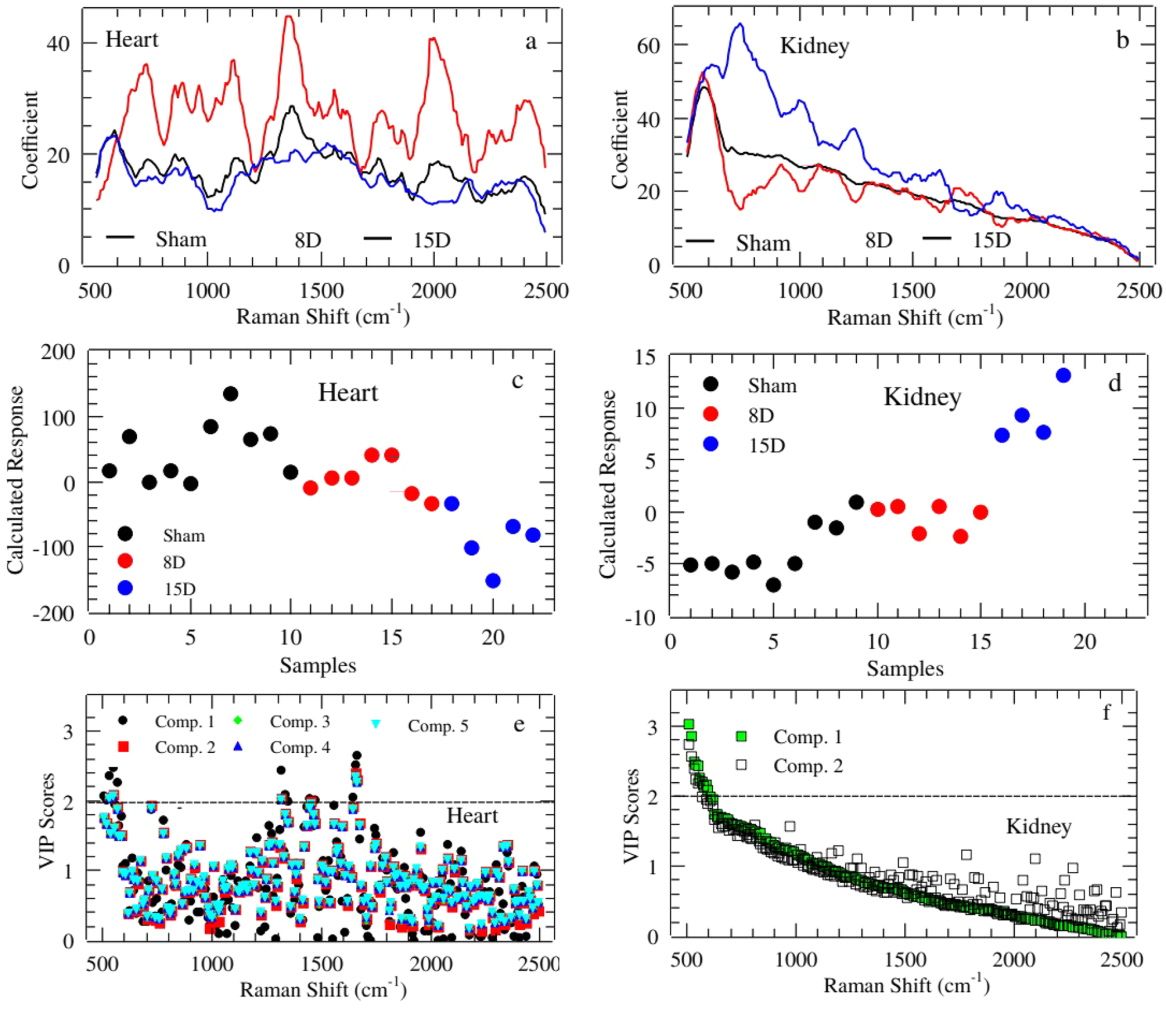
Regression coefficient in PLS-DA for Heart (**a**) and Kidney (**b**). Calculated response for heart (**c**) and kidney (**d**) using up to 5th and 2th component, respectively.Average PLS-DA Variable Importance in Projection (VIP) for those components with best classification performance for Heart (**e**) and Kidney (**f**).

The regression coefficients are shown on Fig. 6 for heart (Fig. 6a) and kidney (Fig. 6b). Both curves appeared smooth not presenting random fluctuations around positive and negative values which would be a symptom of overfitting. However, the coefficients for Sham and 8D groups in heart appeared superimposed in many spectral windows indicating greater similarity among then. The calculated response is shown on Fig. 6(c) and 6(d) for heart and kidney, respectively. Sham and 8D classes appeared confused the most compared to 15D one for heart samples. In fact, the sensibility and specificity for 15D discrimination were 0.80 and 0.94, respectively (see Table 2). Similar trend was observed for kidney.

**Table 2:** Sensibility (S) and Specificity (E) for groups discrimination in heart and kidney samples based on PLS-DA modeling.

	*S*	*E*
**Heart**		
Sham	0.50	0.33
8D	0.14	0.53
15D	0.80	0.94
**Kidney**		
Sham	0.67	0.40
8D	0.00	0.77
15D	1.00	1.00

**Figure 7:**
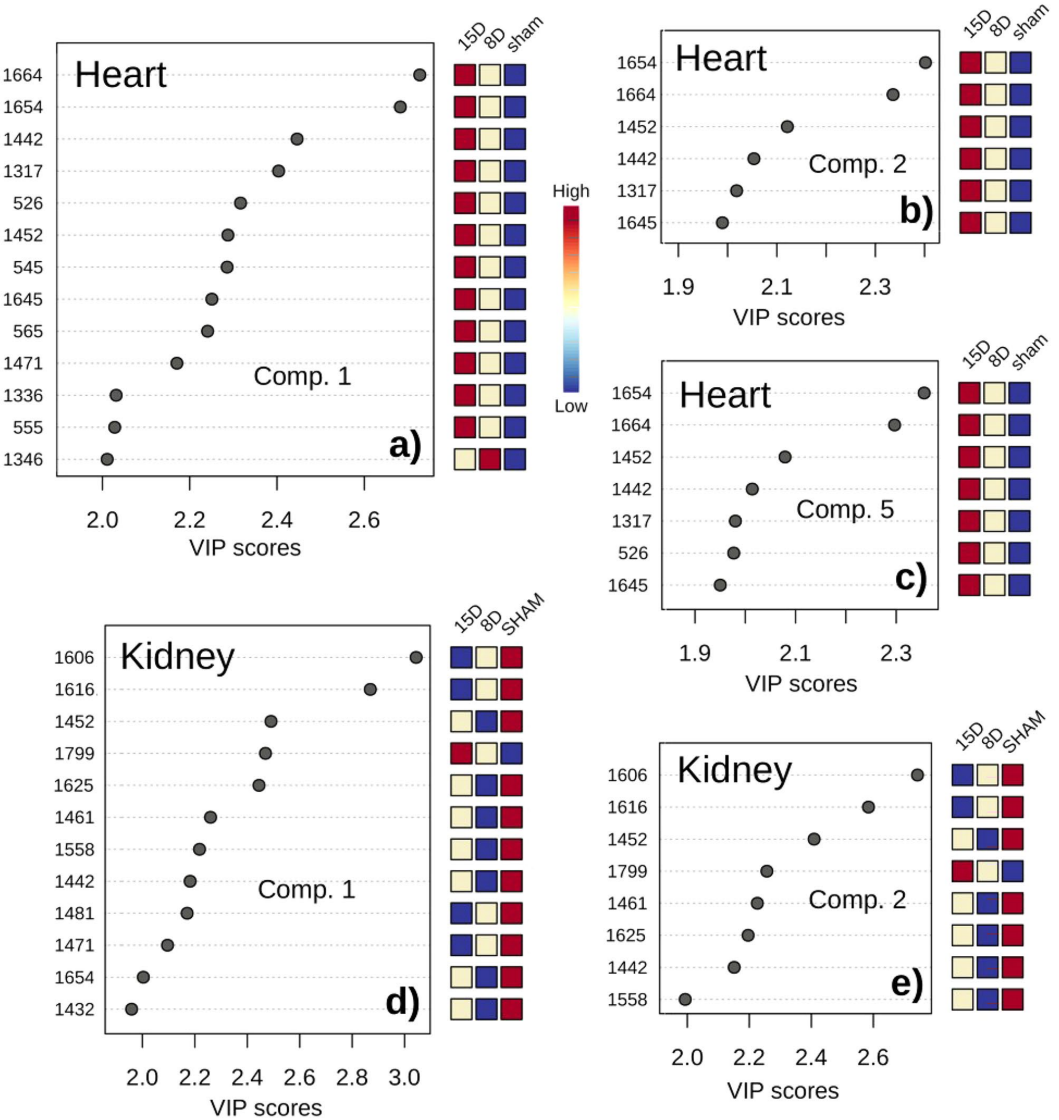
Important vibrational frequencies identified by PLS-DA for heart (components 1, 2, and 5 in **a**, **b**, and **c**, respectively) and kidney (components 1 and 2 in **d** and **e**, respectively). The colored boxes on the right indicate the relative intensity of the corresponding band in each group (Sham, 8D, and 15D).

The important vibrational bands contributing to discrimination are represented on Fig. 7. By observation of increasing and decreasing intensities tendencies along groups and classes we categorized three specific bands trends. A set of bands presented **simultaneous heart and kidney changes**. They were 1442, 1452, 1471, and 1654 cm^−1^ bands that appeared with high intensity for 15D and low intensity for Sham in the heart group. These bands were lower in intensity for 8D being higher for Sham in kidney group. These vibrations are related to collagen and fatty acids. Changes that appeared **only for kidney** correspond to decreasing intensities from Sham to 15D in 1606, 1616, and 1481 cm^−1^ bands (cytosine, tyrosine, Amide II-side chains) and increase from Sham to 15D in 1799 cm^−1^ (lipids). Moreover intensities of 1625, 1461, 1558, and 1432 cm^−1^ bands (tryptophan,  disaccharides, Z DNA) appeared in lower level in 8D. Interestingly, those changes that occurred **exclusively on heart** presented increasing intensities from Sham to 15D in 1664, 1317, 526, 545, 1645, 565, 1336, and 555 cm^−1^ bands (collagen, guanine, cysteine, and α-form of proteins) while 1346 cm^−1^ (glucose) presented higher level in 8D.

## Discussion

We were able to observe that amino acid metabolism was the most perturbed by metabolic pathways in this model of renal ischemia and reperfusion. The discovery of increased tyrosine/tryptophane after 8 and 15 days of reperfusion seems to have important physiological consequences. These amino acids have already been studied in chronic and acute kidney injuries. A tyrosine blood drop has been earlier observed during chronic kidney disease and was explained as an impaired synthesis of tyrosine from phenylalanine, by the kidney^24^. Our results showed that it is possible to identify molecular markers such tyrosine and tryptophane which are the main raw materials of the protein-bounded UTs. The progression of acute to chronic kidney injury is proportional to the quantity of UTs through circulation^25^.

Fibrosis is another relevant physiological point raised on the present study. After 15 days (15D) we have observed an increasing of the band associated to Amide I vibration in heart tissue, related to α-helix secondary structure of collagen. Our previous results indicated the lack of fibrosis in the heart tissue^3^. Physiologically, the non-deposition of collagen does not mean that this gene is not been regulated as well as the increase in some fibroblast growth factors (FGFs). Some of these FGFs are responsible not only for the heart injury but also for the kidney disease progression. One example is FGF23 which is responsible for increase in phosphate levels, aggravating the acute renal injury^26^. FGF23 itself causes hypertrophy left ventricular^27^. Thus we argue that the observed increase in collagen content in cardiac tissue is related to hypertrophic factors activated by collagen but without its deposition on extracellular matrix,  which were evaluated at this time points.

In kidney tissue, after 15 days (15D) we find a decreasing in the band associated to collagen III (cysteine and cistine amino acids bands) in the tissue. Renal IR main consequence is renal fibrosis^28^. Type III collagen is secreted by fibroblasts and other mesenchymal cell types, thus making it a major player in various inflammation-associated pathologies as, e.g., kidney fibrosis^29^. Its serum concentration usually increases in the fibrosis. Thus, collagen III content in the tissue is expected to decrease.

The inflammation in the left kidney is already target of our studies^5^. The inflammation in this model of IR is possibly started by the oxidative stress and enlarged by the NO release, already observed after 8 days of reperfusion comparing data of protein oxidation and superoxide dismutase levels^30^. Oxidative stress is the balance between production of free radicals and reduced antioxidant defenses. It is often increased by inflammation and mitochondrial dysfunction^31^. The oxidative stress works like a gear, activating the transforming growth factor β1 (TGF-β1)^31^ that are able to induce fibrosis in the kidney in part by activating NADPH oxidases^32^. A better understanding of the signaling pathways by which oxidative stress induces renal fibrosis may lead to the development of novel therapeutic strategies.

Trentin-Sonoda et al.^5^ pointed-out an atrophy of the left kidney after IR, based on kidney morphometric data and magnetic resonance imaging. Kidney weight starts to decrease around the 8th day of reperfusion and persists until 15th day and remains smaller even after 8 weeks of reperfusion (data not published). However, the opposite happens to the inflammatory factors: it normalizes after 8 days of IR, as well as the kidney function. Thus, it probably means that kidney function can be normalized once there is a contra-lateral kidney working normally (right kidney) but the injured kidney (left kidney submitted to occlusion) may have a scar due to the fibrosis process induced by the ischemic injury. In a chronic kidney insufficiency or aging, for example, it is possible to observe that normal kidney may be overloaded and unable to maintain control of hemodynamic balance, leading to other cardiac changes. In this sense, collagen in the kidney combined to cysteine and cystine amino acids changes promised be important markers of kidney injury, and predictors of cardiovascular changes observed in CRS 3.

## Conclusions

Our results shown that intensities of vibrations associated to stretching of S–S in cysteine amino acid, stretching of C–C in lipids, twisting of CH_2_ in collagen and phospholipids, bending modes of CH_3_ in lipids and amino acids side chains, Amide I vibration of proteins, are modulated during CRS type 3 induced "in vivo" by renal ischemia and reperfusion. Tyrosine, tryptophan, cystine/cysteine, fibroblast growth factors, and collagen III alterations from homeostasis were the metabolites changes associate with these vibrational changes. These findings are clinically relevant once these bands can be used as molecular markers related to cardiac diseases development in patients with renal injury. Finally, the molecular signatures found as markers of uremic compounds can represent a major clinical finding for acute or chronic kidney diseases and consequently predict future heart diseases.
